# The Value of Salicylic Acid in the Treatment of Rheumatic Diseases of the Eye

**Published:** 1880-05

**Authors:** F. C. Hotz

**Affiliations:** Chicago


### Article V.

### The Value of Salicylic Acid in the Treatment of Rheumatic Diseases of the Eye. By F. C. Hotz, m.d., Chicago.

The beneficial influence of salicylic acid and its salts upon rheumatism has been so often attested that it would be superfluous to write another line about its anti-rheumatic action. But, judging from the scarcity of published reports and from my own observations of practice in eye clinics, I am inclined toward the belief that in the treatment of rheumatic affections of the eye, the salicylates have not yet won the general recognition they justly deserve. The local changes in the eye receive such exclusive attention that the influence of constitutional disorders is often overlooked. In the treatment of iritis, particularly, we are accustomed to rely upon the effect of local medication to such a degree that the use of constitutional remedies is seldom suggested unless syphilis is manifest or suspected.

I would not have it understood that I do not regard these local remedies of paramount importance. I admit that many cases of acute iritis have been cured by the use of atropine together with the proper care of the eye. But occasionally we meet with a case which does not seem to do well, although the atropine develops its full effect upon the iris; the pupil is completely dilated; there are no adhesions between the iris and the anterior capsule of the lens; and still the inflammatory symptoms persist in undiminished severity and the pain about the eye does not abate. It is in such cases that a more careful inquiry may often disclose some constitutional disorder and the use of proper constitutional remedies will be of great advantage.

I will illustrate this point by the following two cases:

1. *Acute rheumatic iritis, severe supra-orbital neuralgia, readily relieved by salicylic acid.*

Geo. W. A——, æt. 42, engineer, presented himself, October 6, 1877, at the Illinois Charitable Eye and Ear Infirmary with all the symptoms of an acute iritis of his right eye, to wit, intense peri-corneal injection, discoloration of the iris, contraction of the pupil, posterior synechiæ and violent supra-orbital pain. The patient had never been troubled with sore eyes, never had syphilis, but had several attacks of rheumatism. His eye had been inflamed two weeks.

Oct. 20.—Although all the posterior synechiæ are broken up and the pupil fully dilated by atropine, the pericorneal redness and supra-orbital neuralgia persist; the pain being particularly violent toward night. Salicylic acid, (gr. iij) 0.25 every two hours.

Oct. 27.—After he had taken ten powders, his head felt quite relieved; and he has been free from pain ever since. Pericorneal injection greatly reduced.

Oct. 30.—No return of the neuralgia; eye free from all inflammation.

2. *Acute Iritis; persistent neuralgia readily relieved by salicylate of soda.*

John McG——, æt. 24, sailor, presented himself at the dispensary of the Eye and Ear Infirmary on February 23d. He had been treated at some other dispensary for an acute iritis of his right eye; but, though the pupil was fully dilated by atropine, the eye was itensely red and painful, the pain being particularly severe at night. Atropine was continued and salicylate of soda given internally.

Feb. 25.—Had no pain last night.

Feb. 28.—Eye continues to improve.

March 15.—Cured.

It seems to me the history of these cases forces upon us the conviction that the earlier administration of the salicylates would have materially shortened the duration of the malady. I had been so impressed by these observations that ever since I have been in the habit of prescribing salicylate of soda in acute iritis when I could discover or suspect a rheumatic diathesis in the patient, just as we would give mercury in iritis when we have reason to believe it to be the local manifestation of constitutional syphilis. And I believe that by this practice iritis has been arrested in its incipiency in some cases and perceptibly shortened in others.

The following cases are instances of this kind.

3. *Acute iritis of left eye; rheumatic history; recovery in one week.*

Mr. H. B——, æt. 50 years, saloon-keeper, had ten years ago a severe attack of articular rheumatism followed by an inflammation of the left eye. This inflammation lasted four weeks, but finally passed away without injuring the sight. On February 21st he came to my office, stating that two weeks previously his left eye became red, painful and sensitive to light; the pain varied a good deal sometimes being quite severe, sometimes scarcely perceptible, but the intermissions did not show any regular periodicity. I noticed a marked redness around the cornea, iris somewhat discolored and dull, pupil small and ragged under the influence of atropine, owing to numerous posterior synechiæ. By the continued use of atropine and the internal administration of sod. salicylate (gr. ijss) (0.20 every two hours) the posterior synechiæ were quickly broken up, and the eye recovered so fast that on February 28th there was not a trace of inflammation left; pupil was clear and regular, iris bright, and no pericorneal redness.

4. *Incipient rheumatic iritis arrested by salicylate of soda.*

Mr. B. P. Q——, æt. 27, traveling clerk, has had several attacks of rheumatism and is very liable to catch cold. Four days ago, while traveling, he stepped from the overheated coach upon the platform in order to cool off. The air was very damp and chilly, and he attributed to that exposure his present trouble. For the next day his left eye became red, painful and sensitive to light. On March 4th, when he consulted me, I found all the symptoms of an incipient iritis (pericorneal redness, contraction of pupil, slight discoloration of the iris, lachrymation, photophobia and pain). I prescribed atropine for the eye and, on account of the rheumatic diathesis, salycilate of soda, (gr. iij) 0.25 every two hours. Two days afterward the patient called again. He had taken twelve powders when the buzzing in his head made him discontinue their use. But his eye had improved so rapidly that it was practically well at this second visit. The pupil was regularly and fully dilated, the iris clear and bright, the pericorneal redness had disappeared and lachrymation and pain subsided.

The instances here reported are selected from a number of similar observations. I preferred to relate a few specimen cases, rather than to fatigue the readers by a long series of cases, because, in view of the well-established value of the salicylates as anti-rheumatic remedies, these few cases are sufficient evidence to show that the salicylates can be as valuable agents in ophthalmic practice as they are in the hands of physicians. For the same purpose I will add two cases of rheumatic paralysis of the ocular muscles, in which the salicylates affirmed their anti-rheumatic power.

5. *Complete paralysis of the right oculo-motor nerve ; rheumatic origin ; recovery by salicylic acid.*

Mrs. D——, æt. 29, came under my care at the dispensary of the Eye and Ear Infirmary on September 15, 1877. She had repeatedly suffered from rheumatic inflammation of the knee-joints and from neuralgia of the forehead. Four weeks ago she was seized with a very violent attack of neuralgia over the right eye ; it lasted three days and when it subsided the right upper eyelid drooped so much that she could not see out of that eye. This condition persisted without change, although she tried a variety of remedies. I found complete ptosis of the right upper lid, due to paralysis of the levator muscle ; and upon raising the paralyzed eyelid, I observed a marked degree of divergent strabismus of the right eye. And it was easily shown that this divergent strabismus was caused by paralysis of the internal rectus muscle, for the patient was unable to turn her right eye toward the left side. In fact, the eye was almost immovably fixed in its position, with the cornea turned toward the external canthus because not only the internus but also all other ocular muscles (superior and inferior rectus, inferior oblique and sphincter pupillæ) dependent upon the third (oculo-motor) nerve were paralyzed.

The patient was at once put upon salicylic acid, (gr. iij) 0.25 three times daily ; and by the 30th of September the muscles of her right eye had recovered so much that she could open it and turn it in every direction just as well as the left eye.

6. *Rheumatic paralysis of the motor-oculi nerve; rapid recovery by salicylic acid.*

Mrs. Carrie Cl——, æt. 32, consulted me on October 10, 1877. Two weeks previously she had stood in the draught of the damp, cold air while her head was wet with perspiration. On the following day she had a violent neuralgia in the left side of her head, lasting three days. When it subsided the sight of her left eye became dim; then she saw everything double and finally the left upper lid dropped down over the left eye so that she could not see out of it at all. This condition had lasted ten days without any perceptible change.

I found complete ptosis of the left upper eyelid; complete dilatation and immobility of the pupil and complete inactivity of the superior, inferior and internal recti and the inferior oblique muscles. In other words, all the ocular muscles supplied by twigs of the oculo-motor nerve were paralyzed. Quinine and salicylic acid, (gr. ij) 0.15 of each, was ordered to be taken every three hours.

Oct. 22d.—The left upper lid can be elevated quite well; left pupil contracting, though still slightly larger than the pupil of the right eye. A faint trace of contractility in the superior and inferior recti.

Oct. 27th.—Marked improvement in the action of the upper and lower recti; only the internus is still totally inactive.

Nov. 7th.—To-day the internus shows a decided improvement. The eye can now be turned in every direction but its rotation toward the right side is still insufficient.

After this date the patient discontinued attendance, so that I have not any direct evidence of her complete recovery. But the fact that she did not return after having improved so fast, may be accepted as a reasonable ground for believing in her recovery.

